# Knowledge of HIV transmission during pregnancy among women of reproductive age in Ghana

**DOI:** 10.1186/s12879-024-09325-w

**Published:** 2024-05-21

**Authors:** Hidaya Mohammed, Martha Suntah Kebir, Comfort Obiribea, Mainprice Akuoko Essuman, Bright Opoku Ahinkorah

**Affiliations:** 1 Department of Microbiology and Immunology, School of Medical Sciences, College of Health and Allied Sciences, University of Cape Coast, Cape Coast, Ghana; 2 Department of Medical Laboratory Science, School of Allied Health Sciences, College of Health and Allied Sciences, University of Cape Coast, Cape Coast, Ghana; 3 Department of Biological Sciences, Southern Illinois University Edwardsville, Edwardsville, IL USA; 4 REMS Consultancy Services, Sekondi-Takoradi, Western Region Ghana; 5 School of Clinical Medicine, University of New South Wales Sydney, Sydney, Australia

**Keywords:** HIV, Knowledge, Mother-to-child transmission, Women, Ghana

## Abstract

**Introduction:**

Human immunodeficiency virus (HIV) remains a significant health challenge affecting many people including those from sub-Saharan Africa (SSA). Even though HIV can be transmitted through various means, mother-to-child transmission (MTCT) remains the major route of transmission in children under the age of five. This study examined the correlates of knowledge of HIV transmission during pregnancy among reproductive-age women in Ghana.

**Methods:**

Data for this study were obtained from the 2014 Ghana Demographic and Health Survey. The sample consisted of 9,106 women aged 15 to 49 years. We conducted both descriptive and multivariable logistic regression analyses to determine the prevalence and factors associated with knowledge of HIV transmission during pregnancy. The results were presented using frequencies, percentages, and adjusted odds ratios (aOR) with their corresponding 95% confidence intervals (CI).

**Results:**

Approximately, 69.41% of women of reproductive age knew of HIV transmission during pregnancy. Women who had two (aOR = 1.32, 95% CI [1.01, 1.72]) or three (aOR = 1.37, 95% CI [1.07, 1.76]) births were more knowledgeable of HIV transmission during pregnancy. Women who read the newspaper (aOR = 1.56, 95% CI [1.31, 1.86]), listened to the radio (aOR = 1.23, 95% CI [1.05, 1.45]), lived in rural areas (aOR = 1.30, 95% CI [1.09, 1.54]) or ever been tested for HIV (aOR = 1.20, 95% CI [1.05, 1.37]) were more likely to be knowledgeable of HIV transmission during pregnancy than their counterparts in the reference categories. Compared to those in the Western Region, women in the Upper East (aOR = 0.45, 95% CI [0.32, 0.63]), Upper West (aOR = 0.54, 95% CI [0.35, 0.85]), Ashanti (aOR = 0.75, 95% CI [0.58, 0.97]) and Greater Accra Regions (aOR = 0.74, 95% CI [0.56, 0.98]) were less knowledgeable of HIV transmission during pregnancy.

**Conclusions:**

The study highlights a gap in the knowledge of HIV transmission during pregnancy among women in Ghana. Continuous public education is required to educate women on HIV transmission from mothers to their children during pregnancy and how this may be interrupted. Such programs should involve the use of the media and take into consideration the demographic and geographic characteristics highlighted as determinants in this study. This will ultimately contribute to the reduction of MTCT of HIV in Ghana.

## Introduction

Despite numerous efforts made towards the elimination of human immunodeficiency virus (HIV) and its associated problems, HIV infection remains a significant global health challenge. In 2021, the global HIV burden was 38.4 million, with approximately 1.5 million new infections and 650,000 deaths [1]. Sub-Saharan Africa (SSA) continues to bear the highest burden, accounting for 67% of the global HIV cases [2]. HIV is transmitted through infected bodily fluids including blood, seminal fluid, vaginal secretions, and breast milk [3–5]. While the virus can be transmitted in a variety of ways, mother-to-child transmission (MTCT) remains the primary mode of infection in young children, especially those under the age of five [5]. MTCT, also known as vertical transmission of HIV, refers to the transmission of the virus from an infected mother to her child and can occur during pregnancy, childbirth, or through breastfeeding [6]. However, the initial potential exposure of the fetus to the virus typically occurs during pregnancy.

HIV infection is associated with diverse range of pregnancy outcomes [7]. The infection, during pregnancy, can result in severe consequences for the developing fetus including neurodevelopmental complications, low birth weight, low Apgar score, and preterm delivery [8, 9]. Furthermore, in the absence of appropriate intervention, there is a 5–10% risk of virus transmission to the fetus particularly during the second and third trimester of pregnancy [10, 12]. The virus has the tendency to traverse the placenta and infect the fetus [11] with high maternal viral load being the most prominent risk factor influencing this transmission mode [12].

Ghana, like many developing countries, is confronted by the global HIV/AIDS epidemic. In 2022, Ghana reported an HIV prevalence of 1.7% with an estimated HIV population of 330,000 adults and 25,000 children [13]. Moreover, the prevalence of HIV among women aged 15 to 49 was 2.4%, which is more than twice of that of men in the same age range [16]. HIV prevalence among pregnant women in Ghana is a matter of profound concern. To illustrate, the prevalence of HIV among pregnant women in the Upper East Region was reported to be 2.1% in 2019 and within the same year, the prevalence among antenatal care attendees in Navrongo stood at 2.0% [5]. This high HIV prevalence among pregnant women and those of childbearing age has major implications for mother-to-child transmission (MTCT) of HIV, as an HIV-infected woman can transmit the virus to her child. Ghana ranks fourth among the 23 high-burden countries identified by UNAIDS in terms of MTCT of HIV, and the second highest in West Africa [14]. It is therefore important to implement effective strategies to reduce the MTCT of HIV and further combat the spread of the virus in the country.

Evidence from HIV-positive Children’s Early antiretroviral treatment studies implies that with efficient implementation of MTCT prevention programs, new infections among infants could be decreased from 40% to less than 5% [15]. The knowledge of MTCT of HIV is paramount in the elimination of MTCT and the effective implementation of prevention of mother-to-child (PMTCT) interventions such as antenatal HIV testing and counseling, and administration of suitable antiretroviral treatment for mothers and newborns [16]. Studies conducted in many countries in SSA have revealed the importance of women’s MTCT knowledge in eliminating pediatric HIV infection [17, 18]. Again, comprehensive maternal knowledge of HIV elimination of MTCT has been demonstrated to be positively linked with maternal HIV testing and antiretroviral therapy (ART) adherence [29, 30]. This is because pregnant women who have good knowledge of HIV and MTCT are more likely to seek testing and treatment to protect themselves and their families from the infection. One million deaths and 2.2 million HIV infections have been prevented in children due to the improvement in knowledge of pregnant women about PMTCT, underscoring the crucial importance of MTCT and PMTCT awareness [19].

Knowledge of reproductive age women on the prevention of MTCT plays a major role in reducing the number of children who are infected by HIV, emphasizing that maternal awareness of MTCT is an effective approach to reducing vertical transmission of HIV [20]. Given that reproductive women are the most vulnerable to HIV and have the potency to pass on the virus to their children during pregnancy, childbirth, and breastfeeding, it is important to educate them regarding the virus transmission and subsequently, appropriate preventive measures. Unfortunately, very little is known about Ghanaian women’s knowledge of HIV transmission during pregnancy which is one of the key mechanisms of MTCT. In this study, we examined the characteristics of women who participated in the 2014 demographic and health surveys to examine the correlates of knowledge of HIV transmission during pregnancy among reproductive-age women in Ghana.

## Methods

### Study setting

The study setting was Ghana. Ghana is a West African country, located about 750 km north of the equator. Ghana is surrounded by three francophone countries: to the west is Cote D’Ivoire, to the east is Togo, and to the north is Burkina Faso. To the south is the 560 km Atlantic Ocean known as the Gulf of Guinea, which forms Ghana’s coastline. Ghana has a land area of about 238,537 km^2^ (92,100 sq. miles). Results from the 2021 Population and Housing Census highlight a shift in Ghana’s population dominant age group from children (0–14 years) to young people aged 15–35. The census revealed that from the year 2000 to 2021, the percentage of young people has increased from 34.6 to 38.2% [21]. The prevalence of HIV in Ghana is dependent on age, geographical location, and gender.

### Data source

This study utilized data from the 2014 Ghana Demographic and Health Survey (GDHS). The GDHS is a survey of data on an array of health-related issues, including HIV/AIDS. Other health issues necessary for the attainment of the Sustainable Development Goals (SDGs) were included in the GDHS survey. The survey was conducted by the Ghana Health Service (GHS) with support from the Ghana Statistical Service (GSS) and ICF International. A multistage sampling method was employed to sample respondents for the survey. The first stage of the sampling involved the selection of clusters from Enumeration Areas (EAs) used for the Population and Housing Census. Next, households were sampled from each cluster and individuals in the sampled households were interviewed. The dataset is openly available and can be accessed at https://dhsprogram.com/data/available-datasets.cfm. This study used 9,106 reproductive-aged women (15–49 years) out of 9,396 women who were interviewed by the DHS. Persons with missing data were excluded from the study. Details on the pre-testing, methods, sampling design selection, and on-site staff training can be found in the GDHS final report.

### Study variables

#### Outcome variable

The outcome variable for this study was knowledge of HIV transmission during pregnancy among women of reproductive age. The question “Can HIV be transmitted from a mother to her baby during pregnancy?” was used to generate information on the outcome variable. Women responded by indicating “Yes”, “No” or “I don’t know”. A dichotomous response was coded. ‘Yes’ was coded ‘1’ when a woman reported that she knew about HIV transmission during pregnancy and ‘No’ or “I don’t know” was coded ‘0’. For this study, a woman is said to be knowledgeable of HIV transmission during pregnancy if she said “Yes” to the question but not knowledgeable if she responded “No” or “I don’t know” to the question.

#### Explanatory variables

The study included fourteen explanatory variables which were selected based on their availability in the dataset and inferences from previous studies [12, 17, 22]. The explanatory variables include the age of the respondent (15–19, 20–24, 25–29, 30–34, 35–39, 40–44, 45–49), marital status (Never married, Married, Cohabitation, Other), highest educational level (No formal education, Primary, Secondary+), religion (Christianity, Islam, Other), total children ever born (Zero birth, One birth, Two births, Three or more births), exposure to TV (No, Yes), exposure to radio (No, Yes), and exposure to the newspaper (No, Yes). Others were ever had sex (No, Yes), ever been tested for HIV (No, Yes), currently pregnant (No/unsure, Yes), wealth index (Poorest, Poorer, Middle, Richer, Richest), place of residence(Urban, Rural), and region (Western, Central, Greater Accra, Volta, Eastern, Ashanti, Brong Ahafo, Northern, Upper East, Upper West). We utilized the existing coding as found in the DHS for age, wealth index, place of residence, pregnancy status, ever been tested for HIV and region. The remaining variables including marital status, level of education, total children ever born, religion, ever had sex and exposure to television, radio and newspaper were recoded because of the small sample sizes of some of their categories.

### Statistical analysis

The data was analyzed using STATA version 14. Descriptive analysis was used to describe the study sample which was also weighted. Descriptive analysis was performed on each variable and the proportion of women with knowledge of HIV transmission during pregnancy was determined. The association between knowledge of HIV transmission during pregnancy and the explanatory variables were determined using the chi-square test for independence. Variables significant at *P* < 0.05 in the chi-square test were selected for a multivariable logistic regression analysis. The multivariable regression analysis was used to determine the factors associated with knowledge of HIV transmission during pregnancy. The results were presented as adjusted odds ratios (aOR) with 95% confidence intervals (CIs). The generalizability of the findings, together with the complex nature of the survey, was accounted for using the Stata command survey set (svy) taking into consideration weight, cluster, and strata. In the analysis, *p*-values less than 0.05 were considered statistically significant.

### Ethical consideration

A formal ethical clearance was not obtained for the study because of the public availability of the DHS dataset. Nonetheless, approval was needed to obtain and use the MEASURE DHS dataset. Permission was granted after the registration of the research project, provision of a description of the data analysis and how the data will be used were submitted through an application. Ethical standards and detailed information about DHS data usage are accessible at https://dhsprogram.com/methodology/Protecting-the-Privacy-of-DHS-Survey-Respondents.cfm.

## Results

### Socio-demographic characteristics of respondents

This study included a sample of 9,106 women with ages ranging from 15 to 49 years. Table 1 shows the socio-demographic characteristics of the study participants. Among the respondents, 17.20% (1,567) of them were aged 20 to 24. Close to half (42.22%) were married. More than half (64.33%) of the women had attained a secondary or a higher level of education. A greater number (80.53%) of the women were Christians and less than half (40.37%) had three or more births. Among the respondents, 80.67% were not exposed to newspapers. However, a higher proportion (77.71% and 85.14%) of the women were exposed to television and radio respectively. The majority of women (87.55%) have had sexual intercourse. In addition, 50.29% had never been tested for HIV and 92.91% were not pregnant or were unsure of their pregnancy status at the time of the survey. The majority of the women were in the richest wealth index (23.83%), urban residents (54.64%), and stayed in either the Greater Accra (20.61%) or Ashanti (19.55%) regions. 

### Distribution of the prevalence of knowledge of HIV transmission during pregnancy across the explanatory variables

Table 1 shows the proportion of women with knowledge of HIV transmission during pregnancy. The results show that 69.41% knew of HIV transmission during pregnancy while the remaining 30.59% did not know of HIV transmission during pregnancy. In the bivariate analysis, age (*p* = 0.001), marital status (*p* < 0.001), total children ever born (*p* < 0.001), exposure to radio (*p* = 0.023), exposure to newspaper (*p* = 0.011) ever had sex (*p* < 0.001), ever been tested for HIV (*p* = 0.003), wealth index (*p* < 0.001), place of residence (*p* < 0.001) and region (*p* < 0.001) were significantly associated with knowledge of HIV transmission during pregnancy.

**Table 1:** Weighted distribution of knowledge of HIV transmission during pregnancy among reproductive-age women across explanatory variables

Variable	Frequency		HIV transmission during pregnancy		*P*-value
	Weighted N	Weighted %	Not knowledgeable (%) 30.59	Knowledgeable (%) 69.41	
**Age of respondent**					0.001
15–19	1556	17.08	34.08 [30.91,37.39]	65.92 [62.61,69.09]	
20–24	1567	17.20	33.37 [30.80,36.73]	66.30 [63.27,69.20]	
25–29	1555	17.08	30.62 [27.70,33.70]	66.38 [66.30,72.30]	
30–34	1332	14.63	31.10 [27.89,34.50]	68.90 [65.50,72.11]	
35–39	1258	13.81	27.66 [24.59,30.97]	72.34 [69.03,75.41]	
40–44	1010	11.09	27.65 [24.23,31.35]	72.35 [68.65,75.77]	
45–49	829	9.11	25.33 [21.78,29.25]	74.67 [70.75,78.22]	
**Marital status**					< 0.001
Never married	2998	32.92	34.64 [32.31,37.04]	65.36 [62.96,67.69]	
Married	3844	42.22	28.43 [26.39,30.56]	71.57 [69.44,73.61]	
Cohabitation	1305	14.33	27.43 [24.50,30.56]	72.57 [69.44,75.50]	
Other	959	10.53	30.91 [27.33,34.74]	69.09 [65.26,72.67]	
**Highest educational level**					0.054
No formal education	1638	17.99	27.35 [24.27,30.67]	72.65 [69.33,75.73]	
Primary	1610	17.68	30.83 [28.11,33.69]	69.17 [66.31,71.89]	
Secondary+	5858	64.33	31.43 [29.63,33.29]	68.57 [66.71,70.37]	
**Religion**					
Christianity	7334	80.53	30.43 [28.84,32.06]	69.57 [67.94,71.16]	0.392
Islam	1379	15.14	32.53 [27.91,37.51]	67.47 [62.49,72.09]	
Other	394	4.32	26.91 [21.00,33.78]	73.09 [66.22,79.00]	
**Total children ever born**					< 0.001
Zero birth	2844	31.23	35.69 [33.31,38.14]	64.31 [61.86,66.69]	
One birth	1299	14.26	31.40 [28.35,34.61]	68.60 [65.39,71.65]	
Two births	1287	14.14	29.17 [26.02,32.53]	70.83 [67.47,73.98]	
Three or more births	3676	40.37	26.86 [24.90,28.92]	73.14 [71.08,75.10]	
**Exposed to TV**					0.687
No	2030	22.29	31.11 [28.33,34.03]	68.89 [65.97,71.67]	
Yes	7076	77.71	30.44 [28.68,32.26]	69.56 [67.74,71.32]	
**Exposed to radio**					0.023
No	1353	14.86	34.05 [30.57,37.72]	65.95 [62.28,69.43]	
Yes	7753	85.14	29.99 [28.41,31.62]	70.01 [68.38,71.59]	
**Exposed to newspaper**					0.011
No	7346	80.67	31.45 [29.76,33.19]	68.55 [66.81,70.24]	
Yes	1760	19.33	27.01 [24.06,30.19]	72.99 [69.81,75.94]	
**Ever had sex**					< 0.001
No	1133	12.45	36.44 [33.06,39.96]	63.56 [60.04,66.94]	
Yes	7973	87.55	29.76 [28.15,31.42]	70.24 [68.58,71.85]	
**Ever been tested for HIV**					0.003
No	4580	50.29	32.52 [30.64,34.46]	67.48 [65.54,69.36]	
Yes	4526	49.71	28.64 [26.58,30.79]	71.36 [69.21,73.42]	
**Currently pregnant**					0.214
No/unsure	8460	92.91	30.80 [29.19,32.46]	69.20 [67.54,70.81]	
Yes	646	7.09	27.83 [23.66,32.43]	72.17 [67.57,76.34]	
**Wealth index**					< 0.001
Poorest	1365	14.99	29.81 [26.37,33.49]	70.19 [66.51,73.63]	
Poorer	1583	17.38	26.05 [23.27,29.03]	73.95 [70.97,76.73]	
Middle	1902	20.89	28.87 [26.19,31.70]	71.13 [68.30,73.81]	
Richer	2087	22.92	30.97 [28.17,33.92]	69.03 [66.08,71.83]	
Richest	2170	23.83	35.54 [32.20,39.03]	64.46 [60.97,67.80]	
**Place of residence**					< 0.001
Urban	4976	54.64	33.83 [31.66,36.08]	66.17 [63.92,68.34]	
Rural	4130	45.36	26.68 [24.72,28.75]	73.32 [71.25,75.28]	
**Region**					< 0.001
Western	1009	11.08	25.77 [22.04,29.89]	74.23 [70.11,77.96]	
Central	928	10.19	22.50 [20.19,24.99]	77.50 [75.01,79.81]	
Greater Accra	1877	20.61	35.46 [31.54,39.59]	64.54 [60.41,68.46]	
Volta	673	7.39	29.73 [25.40,34.44]	70.27 [65.56,74.60]	
Eastern	859	9.44	31.17 [27.16,35.48]	68.83 [64.52,72.84]	
Ashanti	1780	19.55	33.74 [30.19,37.48]	66.26 [62.52,69.81]	
Brong Ahafo	744	8.17	26.90 [22.45,31.87]	73.10 [68.13,77.55]	
Northern	681	7.48	21.83 [16.65,28.09]	78.17 [71.91,83.35]	
Upper East	345	3.79	44.28 [38.75,49.95]	55.72 [50.05,61.25]	
Upper West	210	2.30	38.75 [30.32,47.92]	61.25 [52.08,69.68]	

### Determinants of knowledge of HIV transmission during pregnancy

Table 2 displays the results from the multivariable logistic regression analysis of the determinants of knowledge of HIV transmission during pregnancy among Ghanaian women. From the results, women who have two (aOR = 1.32, 95% CI [1.01, 1.72]) or three (aOR = 1.37, 95% CI [1.07, 1.76]) births have higher odds of being knowledgeable about HIV transmission during pregnancy compared to their counterparts without a child. Women who read the newspaper (aOR = 1.56, 95% CI [1.31, 1.86]) and those who listened to the radio (aOR = 1.23, 95% CI [1.05, 1.45]) are more likely to know about HIV transmission during pregnancy than those who were not exposed to these media sources. The odds of knowing about HIV transmission during pregnancy were higher among women who have ever been tested for HIV (aOR = 1.20, 95% CI [1.05, 1.37]) than those who have never been tested for HIV. Women of rural residence (aOR = 1.30, 95% CI [1.09, 1.54]) were more likely to know about HIV transmission during pregnancy than those in urban areas. Compared to those in the Western Region, women from the Upper East (aOR = 0.45, 95% CI [0.32, 0.63]), Upper West (aOR = 0.54, 95% CI [0.35, 0.85]), Ashanti (aOR = 0.75, 95% CI [0.58, 0.97]) and Greater Accra (aOR = 0.74, 95% CI [0.56, 0.98]) regions have lesser odds of being knowledgeable of HIV transmission during pregnancy.

**Table 2:** Multivariable logistic regression analysis of factors associated with knowledge of HIV transmission during pregnancy among women of reproductive age in Ghana.

Variable	Multivariable analysis	*P*-value
	aOR [95% CI]	
**Age of respondent**		
15–19	1	
20–24	0.87 [0.70,1.07]	0.184
25–29	0.94 [0.72,1.22]	0.628
30–34	0.88 [0.67,1.14]	0.336
35–39	1.03 [0.76,1.40]	0.850
40–44	1.06 [0.78,1.44]	0.710
45–49	1.22 [0.84,1.78]	0.287
**Marital status**		
Never married	1	
Married	0.97 [0.78,1.21]	0.801
Cohabiting	1.06 [0.82,1.37]	0.643
Other	0.87 [0.67,1.12]	0.275
**Total children ever born**		
Zero birth	1	
One birth	1.14 [0.93,1.41]	0.200
Two births	1.32 [1.01,1.72]	0.041
Three or more births	1.37 [1.07,1.76]	0.012
**Exposed to radio**		
No	1	
Yes	1.23 [1.05,1.45]	0.010
**Exposed to newspaper**		
No	1	
Yes	1.56 [1.31,1.86]	< 0.001
**Ever had sex**		
No	1	
Yes	1.06 [0.87,1.29]	0.573
**Ever been tested for HIV**		
No	1	
Yes	1.20 [1.05,1.37]	0.008
**Wealth index**		
Poorest	1	
Poorer	1.08 [0.87,1.35]	0.481
Middle	1.02 [0.79,1.32]	0.870
Richer	1.01 [0.77,1.33]	0.926
Richest	0.85 [0.63,1.16]	0.299
**Place of residence**		
Urban	1	
Rural	1.30 [1.09,1.54]	0.004
**Region**		
Western	1	
Central	1.18 [0.92,1.51]	0.181
Greater Accra	0.74 [0.56,0.98]	0.038
Volta	0.77 [0.57,1.06]	0.105
Eastern	0.76 [0.57,1.02]	0.066
Ashanti	0.75 [0.58,0.97]	0.026
Brong Ahafo	0.95 [0.69,1.31]	0.764
Northern	1.31 [0.87,1.97]	0.196
Upper East	0.45 [0.32,0.63]	< 0.001
Upper West	0.54 [0.35,0.85]	0.007

## Discussion

This study investigated the prevalence and factors associated with knowledge of HIV transmission during pregnancy among Ghanaian women of reproductive age. The study’s findings reveal that the majority of the participants (69.41%) were aware that HIV can be transmitted during pregnancy. The observed proportion of women knowledgeable of HIV transmission in pregnancy is higher than those reported in similar studies in Ethiopia and Cameroon [20, 22]. However, some studies conducted on a similar topic within SSA have reported relatively higher proportion of women with good knowledge of HIV transmission in pregnancy [7, 17, 23, 24]. It is worrying to observe that a good number of women in their reproductive years, roughly one-third (30.59%) of the study population, were unaware of the risk of HIV transmission during pregnancy. This lack of awareness among this population poses a threat, as these women, if HIV positive, may inadvertently pass the virus to their vulnerable unborn children, thus potentially adding to the global and national burden of pediatric HIV infections.

The knowledge of HIV transmission during pregnancy was found to be influenced by factors such as HIV testing history, the total number of children ever born, media exposure, place of residence, and geographic location. Women who had ever tested for HIV had higher odds of possessing knowledge of HIV transmission during pregnancy, compared to those who had never taken the test. This can be attributed to the counseling services that usually accompany HIV testing before or after the test. A study conducted among women in Tanzania revealed a similar outcome [25]. With the frequency of childbirth comes an increased engagement with antenatal care services, which encompass educational campaigns focused on maternal transmission of HIV to children and its preventive measures [5]. Given the commendable antenatal attendance rate in Ghana and the elaborate information nurses and midwives give to pregnant women, it comes as no surprise that this study identified a positive association between the number of children a woman has and an increased probability of possessing knowledge about HIV transmission during pregnancy [26].

The influence of media exposure on awareness of HIV transmission during pregnancy has become evident, with different media platforms demonstrating varying degrees of impact. In the present study, women who read newspapers had a higher likelihood of possessing knowledge about HIV transmission during pregnancy than those who do not. Similarly, those who were exposed to radio broadcasts were more likely than those who had limited exposure to radio to be knowledgeable about HIV transmission in pregnancy. This points to the important role radio and print media play in disseminating health-related information. Similar findings were documented in studies involving Ethiopian women [27] and a diverse group of women from various SSA regions [17]. An earlier study acknowledged newspapers as an effective information source that has long been pivotal in raising public awareness about HIV transmission [28]. Educational interventions aimed at educating women on the transmission of HIV should consider making use of these media platforms.

In contrast to findings in other studies [2, 7, 18, 20], this study brought to light an intriguing pattern based on the geographic location of participants. First, women residing in rural areas had higher likelihood of possessing knowledge about HIV transmission during pregnancy compared to those living in urban cities. The possible explanation for this observation is the information dissemination approach in rural areas of Ghana. These rural areas are equipped with information centers that broadcast health-related issues in local dialects, ensuring comprehensive understanding. This contrasts with urban cities, where such extensive outreach may be lacking. Women in the Upper East, Upper West, Ashanti, and Greater Accra regions have a lower likelihood of being informed about HIV transmission during pregnancy, compared to their counterparts in other regions. These geographic disparities in the awareness of HIV transmission may be attributed to variations in the access to awareness campaigns. It is important to identify geographic disparities in the awareness of the transmission of HIV in planning HIV elimination interventions.

### Strengths and limitations

The dataset utilized in this study is considerably large and very representative of the population of Ghanaian women and was collected using standardized methods, thus enhancing the generalizability and reliability of the study. However, the study should be interpreted bearing in mind these limitations. As this study employed a cross-sectional design, it cannot establish causation between the determinants and knowledge of HIV transmission during pregnancy. Additionally, participants’ responses may be influenced by social desirability bias leading to potential inaccuracies in the data. Despite these limitations, the findings of this study can be useful to policymakers and stakeholders, as they offer valuable information on the factors that influence knowledge regarding HIV transmission among reproductive women in Ghana.

## Conclusion

This study illuminates the extent of the knowledge gap among reproductive-aged Ghanaian women regarding HIV transmission during pregnancy, a critical aspect of MTCT. Additionally, the study explores various determinants that influence this knowledge. The study’s outcomes highlight specific determinants that should be prioritized for tailored interventions to bridge the knowledge gap and ensure universal awareness of HIV transmission during pregnancy. These interventions should include the utilization of various media platforms for creating awareness, and addressing geographic and demographic disparities to ensure consistent knowledge across the population. Ultimately, this will contribute to the overarching goal of reducing MTCT in Ghana.

## Data Availability

Data for this study were sourced from Demographic and Health Surveys (DHS) and are available here: http://dhsprogram.com/data/available-datasets.cfm.
